# Real-world treatment patterns and visual outcomes of faricimab in patients with diabetic macular oedema in the UK at 12 months: the FARWIDE-DMO study

**DOI:** 10.1038/s41433-025-04059-8

**Published:** 2025-10-23

**Authors:** Tunde Peto, Ian Pearce, James Talks, Gabriella de Salvo, Praveen J. Patel, Samantha R. de Silva, Richard P. Gale, Sobha Sivaprasad, Deepali Varma, Rhianon Reynolds, Clare Bailey, Louise Downey, Christine Kiire, Gloria C. Chi, Melanie Dodds, Natalee James, Amanda K. Downey, Parul Dayal

**Affiliations:** 1 Queen’s University Belfast and Belfast Health and Social Care Trust, Belfast, UK; 2 Liverpool University Hospitals NHS Foundation Trust, Liverpool, UK; 3 Newcastle Hospitals NHS Foundation Trust, Newcastle upon Tyne, UK; 4 University Hospital Southampton NHS Foundation Trust, Southampton, UK; 5 University of Southampton, Southampton, UK; 6 NIHR Moorfields Biomedical Research Centre, Moorfields Eye Hospital, London, UK; 7 Oxford University Hospitals NHS Foundation Trust, Oxford, UK; 8 Nuffield Department of Clinical Neurosciences, University of Oxford, Oxford, UK; 9 University of York, York, UK; 10 York and Scarborough Teaching Hospitals NHS Foundation Trust, York, UK; 11 South Tyneside and Sunderland NHS Foundation Trust, Sunderland, UK; 12 Aneurin Bevan University Health Board, Newport, UK; 13 Cardiff University School of Vision Science, Cardiff, UK; 14 University Hospitals Bristol NHS Foundation Trust, Bristol, UK; 15 Hull University Teaching Hospital, Hull, UK; 16 Genentech, Inc., San Francisco, CA USA; 17 Medisoft Ltd., Leeds, UK; 18 Roche Products Ltd., Welwyn Garden City, UK; 19 F. Hoffmann-La Roche Ltd., Mississauga, ON Canada

**Keywords:** Outcomes research, Retinal diseases

## Abstract

**Background:**

The Faricimab Real-World Evidence (FARWIDE) studies are evaluating real-world outcomes of eyes with diabetic macular oedema (DMO)/neovascular age-related macular degeneration (nAMD) treated with faricimab in the UK. We present results from FARWIDE-DMO for eyes with 12 months of follow-up after faricimab initiation.

**Methods:**

FARWIDE-DMO includes patients with diagnosis of DMO who received ≥1 intravitreal faricimab injection after May 2022 in the diagnosed eye(s) at 1 of 35 UK National Health Service sites. All eyes had ≥12 months of follow-up after faricimab initiation as of July 2024. Treatment-naïve (TN) eyes had no prior anti-VEGF therapy or steroid implant. Previously treated (PT) eyes switched to faricimab. Baseline characteristics, visual acuity (VA) outcomes and treatment patterns were evaluated. Intraocular inflammation (IOI) and presumed infectious endophthalmitis (PIE) incidences were pooled for all nAMD/DMO eyes with any follow-up duration. Analyses are descriptive.

**Results:**

2147 eyes (1564 patients) were included (TN, 32.1%; PT, 67.9%). For TN eyes, mean (standard deviation [SD]) VA at baseline and 12 months were 63.9 (15.2) and 68.4 (16.3) Early Treatment Diabetic Retinopathy Study letters, respectively. VA remained stable in PT eyes. TN eyes received a mean (SD) of 4.5 (1.0) faricimab injections in months 1–6 and 1.9 (1.2) injections in months 7–12. PT eyes received 4.5 (1.2) injections in months 1–6 and 2.4 (1.3) in months 7–12. IOI and PIE rates were consistent with faricimab phase 3 trials.

**Conclusions:**

These 1-year data support real-world effectiveness, durability and safety of faricimab in DMO.

## Introduction

Anti–vascular endothelial growth factor (VEGF) monotherapy agents administered via regular intravitreal injections have traditionally been the standard of care for people with retinal vascular disorders, including diabetic macular oedema (DMO) [1]. Although these agents lead to visual gains in clinical trials [2, 3], similar gains are not achieved in real-world clinical practice [4]. In real-world settings, patients with DMO receive fewer anti-VEGF injections and exhibit worse visual gains compared with patients in clinical trials [4]. Discrepancies between clinical trials and real-world outcomes has largely been attributed to the treatment burden on patients, caregivers and clinics/healthcare systems associated with chronic, frequent intravitreal injections (which may be as often as every 4 weeks) and regular follow-up visits [5, 6]. Although clinical trial participants are carefully selected, treated and monitored according to study protocols, treatment adherence patterns and outcomes in real-world practice often vary owing to differences in patient demographics, socioeconomic status, disease characteristics, comorbidities, prior treatment history, patient and provider preferences, local treatment guidelines and reimbursement [4, 6–10]. These factors may make sustaining the burden of frequent treatment challenging for patients, which impacts outcomes. This suggests that more durable therapies that reduce the treatment burden associated with retinal vascular disorders are needed, as are studies that evaluate new treatments in real-world practice.

Faricimab is a humanised, bispecific monoclonal antibody approved for treatment of DMO, neovascular age-related macular degeneration (nAMD) and macular oedema secondary to retinal vein occlusion [11–17]. Faricimab addresses the multifactorial nature of these disorders by targeting VEGF to reduce pathological neovascularisation while stabilising retinal blood vessels and reducing inflammation through angiopoietin-2 inhibition [15, 18]. Safety and efficacy of faricimab in eyes with DMO were demonstrated in the pivotal YOSEMITE/RHINE phase 3 trials. Both trials showed robust vision gains and anatomical improvements for faricimab with flexible dosing up to every 16 weeks [15] that were maintained through the second year of treatment [19]. This suggests that faricimab could be used to extend treatment intervals for patients with DMO and reduce their treatment burden.

Although results from the pivotal faricimab clinical trials are supported by observations from a growing number of multinational real-world studies [20–25], most of these studies are small (<200 patient), single-centre studies with limited generalisability and a short follow-up time period (<6 months). There remains a need for larger, more generalisable and longer-term studies to investigate faricimab effectiveness and durability in real-world settings. The ongoing Faricimab Real-World Evidence (FARWIDE) studies evaluate the real-world treatment patterns and outcomes over time in eyes with DMO (FARWIDE-DMO) or nAMD (FARWIDE-nAMD) receiving faricimab in the United Kingdom (UK). This paper describes results from the FARWIDE-DMO study for patients with 12 months of follow-up.

## Methods

### Objectives

The primary objectives of FARWIDE-DMO are to evaluate baseline characteristics, treatment frequency and visual outcomes of faricimab treatment over time amongst patients with DMO in the real-world setting. An exploratory objective was analysis of faricimab safety in both DMO and nAMD eyes. This paper focusses on the results amongst treatment-naïve and previously treated patient eyes with DMO that had ≥12 months of follow-up following their first faricimab treatment.

### Study design and data source

FARWIDE-DMO is a retrospective, observational study. Patient data were extracted from the Medisoft Ophthalmology (first-generation) or mediSIGHT (flagship) electronic medical record (EMR) systems (Medisoft Ltd., Leeds, UK; additional details in the Supplementary Methods) from 35 National Health Service trusts with medical retina clinics across the UK (listed in Supplementary Table 1). Data were extracted and analysed by Medisoft Ltd. Data were anonymised by removing the patient, study site and clinician identifier and by perturbing all dates on the patient’s record by a randomly generated number of days (−180 to +180, excluding zero). Data reported were collected between June 2022 and July 2024.

### Patient population

Patients were ≥18 years of age at baseline, defined as the date of first faricimab injection. Patients had a documented diagnosis of DMO and received ≥1 intravitreal faricimab injection in the diagnosed eye(s) on or after June 2022 (both eyes of a patient were included if they met the criteria). Exclusion criteria included concomitant nAMD or retinal vein occlusion, prior or concomitant participation in any faricimab clinical trials and/or any trials requiring intravitreal injections, and not having a clear record regarding prior anti-VEGF treatments and steroid implants at the time of the first faricimab injection at baseline. Anti-VEGF treatments of interest were aflibercept 2 mg, ranibizumab, brolucizumab, bevacizumab (off-label) and biosimilars. Steroid implant treatments of interest were dexamethasone and fluocinolone acetonide.

### Study variables

#### Baseline patient characteristics

Patient age in years at baseline, sex (male, female, or not stated), race (White British, White Irish, Asian or Asian British, Black or Black British, Chinese, any other White background, any other ethnic group, any other mixed background or not stated) and socioeconomic status were reported. Race was self-reported by the patient. Socioeconomic status was defined by the Index of Multiple Deprivation; a small area measure of relative deprivation across the constituent nations of the UK, detailed in the Supplement and categorised as deciles ranked from most (1) to least (10) deprived.

#### Baseline ocular characteristics

Laterality of DMO disease and faricimab treatment (unilateral or bilateral) was extracted. Counts and percentages of treatment-naïve and previously treated patient eyes were also extracted. Eyes that were not treated with intravitreal anti-VEGF therapy or steroid implant before baseline were considered 'treatment-naïve', whereas eyes treated with anti-VEGF therapy or steroid implant before baseline were considered 'previously treated'. Patients were discontinued from follow-up if they received at least two doses of a non-faricimab treatment or a steroid implant for DMO.

The number of distinct anti-VEGF agents used any time before baseline was categorised as 1, 2, 3 or >3 agents. The anti-VEGF agent used immediately before faricimab and the number of anti-VEGF injections received any time before faricimab initiation were also evaluated.

#### Faricimab treatment characteristics

The number of faricimab injections received in the first 12 months, months 1–6 and months 7–12 of faricimab treatment were evaluated. The number of loading doses received was defined as the number of consecutive faricimab injections that had a treatment interval of 28 ± 14 days, up to the fourth injection. The number of loading doses received was categorised as 1, 2, 3 or 4 injections.

Duration of faricimab follow-up was defined as the time between baseline and the last visit before the data extraction or date of the first non-faricimab treatment after baseline, where present.

#### Visual acuity outcomes

Visual acuity (VA) captured on or within 28 days before the first faricimab injection was considered baseline VA. For the 12-month time point, measurements from a window of 56 days at either side of the 12-month time point after faricimab initiation were included. If multiple measurements were available, the value closest to baseline or 12-month follow-up date was selected. VA measurements were recorded in the patient’s EMR using Early Treatment Diabetic Retinopathy Study (ETDRS) letter score, Snellen score (metres) or logMAR. The Gregori et al. [26] method was used to convert Snellen and logMAR values to approximate ETDRS letters (referred to subsequently as ETDRS letters). VA was grouped into the following categories: ≤34, 35–55, 56–69, ≥70 letters for presentation of baseline VA-stratified results. Attainment and maintenance of VA ≥ 70 letters (driving-level VA) [27] at 12 months were assessed amongst eyes with VA < 70 and ≥70 ETDRS letters at baseline, respectively. Gain or avoidance of loss of 10 ETDRS letters was assessed in eyes with VA of ≤90 and ≥10 ETDRS letters at baseline, respectively. Gain or avoidance of loss of 15 ETDRS letters was assessed in eyes with VA of ≤85 and ≥15 ETDRS letters at baseline, respectively.

### Adverse events

Adverse event (AE) analysis was limited to intraocular inflammation (IOI) and presumed infectious endophthalmitis (PIE). Both IOI and PIE incidences were pooled for nAMD and DMO eyes with any duration of follow-up throughout the FARWIDE studies. Eyes were excluded from this analysis if there was evidence of IOI or PIE in the 12 months before the first faricimab injection. Included AEs were within 180 days after a faricimab injection and before a subsequent non-faricimab treatment. The observation window for an injection-related AE was from the date of the injection until 180 days following a faricimab injection, date of data extraction or the date of next injection administered in the study eye, whichever was earlier. If AEs of the same type occurred within 30 days of each other, the first was included. One event per injection was counted. IOI and PIE events were identified using the post-operative complications, diagnoses or clinical examination findings on the patient record in the EMR [28]. PIE events were additionally identified using surgical records of vitreous biopsy or anterior chamber tap or intravitreal treatment with ceftazidime or vancomycin on the patient’s record in the EMR [28]. We report the rate of events per 100 injections (% of events per injection). The exact χ² method was used to calculate the 95% confidence interval (CI) for the rates. Additional details, including clinical terms used to define IOI and PIE events, are included in the Supplementary Methods.

### Statistical analysis

Analyses are descriptive. Means (standard deviations [SDs]) were reported for continuous variables. Frequency and percentages were reported for categorical variables. Additional details are included in the Supplementary Methods.

### Ethical approvals

The study was conducted in accordance with the Declaration of Helsinki and the UK’s Data Protection Act. The use of de-identified patient data was approved by the Caldicott Guardian and lead clinician for the Medical Retina Service at each site. Patient informed consent was not required.

## Results

### Study population and baseline characteristics

**Fig. 1:**
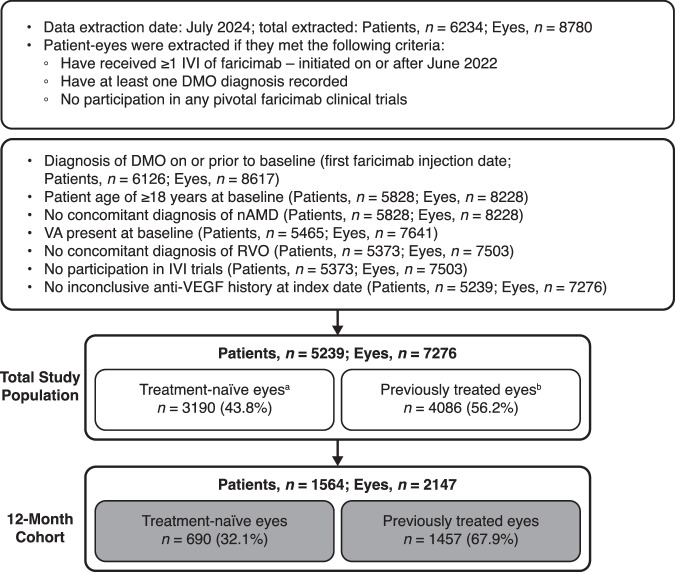
Patient attrition. ^a^Treatment-naïve eyes are those with no evidence of anti-VEGF injections or steroid implant at any time before initiating faricimab. ^b^Previously treated eyes are defined as those that received ≥1 prior anti-VEGF treatment or steroid implant any time before faricimab initiation at the participating site. DMO diabetic macular oedema, IVI intravitreal injection, nAMD neovascular age-related macular degeneration, RVO retinal vein occlusion, VEGF vascular endothelial growth factor.

Between June 2022 and July 2024, 7276 patient eyes (5239 patients) initiated faricimab treatment and met the criteria for the FARWIDE-DMO study, of which 2147 eyes (1564 patients) had ≥12 months of follow-up since initiating faricimab treatment by July 2024. Here, we report results from the patient eyes in this 12-month cohort, where 690 eyes (32.1%) were treatment naïve, and 1457 eyes (67.9%) were previously treated (Fig. 1).

Baseline demographics are shown in Table 1. The mean (SD) age of the cohort at baseline was 62.5 (11.6) years. Most of the patients were male (61.3%), and approximately half were White (52.0%). In total, 25.5% of the treatment-naïve eyes and 22.1% of the previously treated eyes were in the most deprived Index of Multiple Deprivation deciles (1–2) (Table 1).

**Table 1:** Baseline characteristics of the 12-month cohort.

Baseline characteristics	Treatment-naïve eyes (*n* = 690)	Previously treated eyes (*n* = 1457)
Age, mean (SD), years	60.5 (12.2)	62.9 (10.9)
Sex, *n* (%)		
Female	254 (36.8)	534 (36.7)
Male	430 (62.3)	907 (62.3)
Not stated	6 (0.9)	16 (1.1)
Race, *n* (%)		
White/White British/White Irish	347 (50.3)	774 (53.1)
Asian/Asian British	67 (9.7)	103 (7.1)
Black/Black British	20 (2.9)	34 (2.3)
Other^a^	22 (3.2)	46 (3.2)
Not stated	234 (33.9)	500 (34.3)
Index of Multiple Deprivation deciles (34),^b^ *n* (%)		
1–2 (most deprived)	176 (25.5)	322 (22.1)
3–4	148 (21.4)	306 (21.0)
5–6	126 (18.3)	266 (18.3)
7–8	131 (19.0)	268 (18.4)
9–10 (least deprived)	100 (14.5)	249 (17.1)
Unknown	9 (1.3)	46 (3.2)
Laterality of disease, *n* (%)		
Unilateral	96 (13.9)	108 (7.4)
Bilateral	594 (86.1)	1349 (92.6)
Laterality of faricimab treatment, *n* (%)		
Unilateral	291 (42.2)	427 (29.3)
Bilateral	399 (57.8)	1030 (70.7)
Baseline VA, ETDRS letters		
Mean (SD), ETDRS letters	63.9 (15.2)^c^	65.8 (15.3)^d^
Median (Q1, Q3)	66 (55, 76)^c^	70 (58, 76)^d^
Eyes by baseline VA category (ETDRS letter), *n* (%)		
≤34	21 (4.7)^c^	52 (3.9)^d^
35–55	94 (21.1)^c^	259 (19.4)^d^
56–69	122 (27.4)^c^	317 (23.7)^d^
≥70	209 (46.9)^c^	709 (53.0)^d^
**Ocular characteristics at baseline**		
Baseline ocular conditions, *n* (%)		
Glaucoma	24 (3.5)	118 (8.1)
Cataract	147 (21.3)	491 (33.7)
Amblyopia	11 (1.6)	6 (0.4)
Lens status, *n* (%)		
Phakic	478 (69.3)	893 (61.3)
Pseudophakic	129 (18.7)	513 (35.2)
Aphakic	2 (0.3)	0 (0.0)
Not stated	81 (11.7)	51 (3.5)
**Prior anti-VEGF treatment characteristics**		
Duration of anti-VEGF treatment prior to faricimab initiation, years^e^		
Mean (SD)		2.8 (2.3)
Median (Q1, Q3)		2 (1.4)
Total number of anti-VEGF injections before faricimab initiation		
Mean (SD)	–	16.2 (11.7)
Median (Q1, Q3)		13 (7.22)
Number of distinct anti-VEGF agents before faricimab, *n* (%)		
1		1100 (76.7)
2	–	301 (21.0)
≥3		32 (2.2)
Last anti-VEGF treatment received before initiating faricimab, *n* (%)		
Aflibercept 2 mg		1212 (84.6)
Ranibizumab	–	176 (12.3)
Bevacizumab		31 (2.2)
Ranibizumab biosimilars^f^		14 (1.0)

Baseline is defined as date of first faricimab injection.

*ETDRS* Early Treatment Diabetic Retinopathy Study, *LSOA* Lower Layer Super Output Area, *nAMD* neovascular age-related macular degeneration, *Q* quartile, *SD* standard deviation, *VA* visual acuity, *VEGF* vascular endothelial growth factor.

^a^Includes any other ethnic group and any other mixed background.

^b^The deciles are calculated per nation by ranking the 32,844 LSOAs in England and 6976 Data Zones in Scotland from most deprived to least deprived and dividing them into 10 equal groups, respectively. Areas in decile 1 fall within the most deprived 10% and areas in decile 10 fall within the least deprived 10% of LSOAs/Data Zones per nation.

^c^*n* = 1181 for eyes with VA at baseline and 12 months.

^d^*n* = 4577 for eyes with VA at baseline and 12 months.

^e^Number of years between the first anti-VEGF injection and the first faricimab injection.

^f^Ranibizumab biosimilars were Byooviz^TM^ and Ongavia®.

At the patient level, faricimab treatment was bilateral in 57.8% of treatment-naïve and 70.7% of previously treated eyes. Cataract was the most common of the studied baseline ocular conditions (21.3% of the treatment-naïve eyes and 33.7% of the previously treated eyes; Table 1).

Previously treated eyes had been on anti-VEGF treatment for a mean (SD) of 2.8 (2.3) years and received a mean (SD) of 16.2 (11.7) anti-VEGF injections before switching to faricimab treatment. 76.8% of the eyes received only one distinct anti-VEGF agent before switching to faricimab treatment (Table 1). 84.6% of patients received aflibercept 2 mg, 12.3% received ranibizumab, 2.2% received bevacizumab and 1.0% received ranibizumab biosimilar as their anti-VEGF treatment immediately before switching to faricimab (Table 1). 11.7% (*n* = 171) of previously treated eyes had steroid intravitreal injections or implants.

### Faricimab treatment: injection frequency

**Fig. 2:**
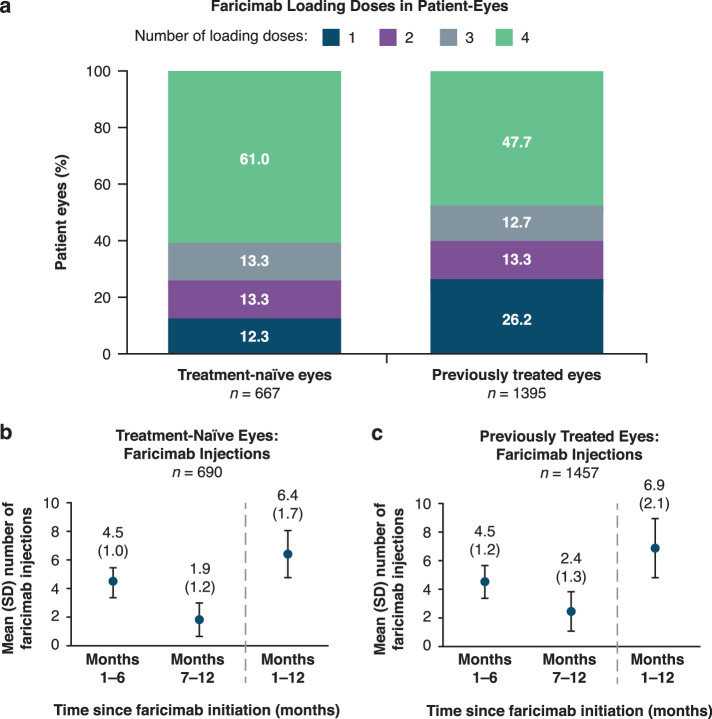
Faricimab loading doses and injection frequency in the 12-month cohort. **a** Number of loading doses received in treatment-naïve and previously treated patient-eyes. **b** Mean (SD) number of faricimab injections in the first and second 6 months of treatment in treatment-naïve eyes. **c** Mean (SD) number of faricimab injections in the first and second 6 months of treatment in previously treated eyes. Total count is amongst eyes with ≥4 faricimab injections. Loading doses were defined as consecutive faricimab injections per patient-eye that have a treatment interval of 28 ± 14 days (14–42 days), up to the fourth injection. SD standard deviation.

Amongst eyes that received at least four faricimab injections (667 treatment-naïve and 1395 previously treated eyes), 61.0% of the treatment-naïve group and 47.7% of the previously treated group received four faricimab loading doses (Fig. 2a). The mean (SD) number of faricimab injections between baseline and month 12 was 6.4 (1.7) for treatment-naïve eyes. These eyes received 4.5 (1.0) faricimab injections in months 1–6 and 1.9 (1.2) injections in months 7–12 (Fig. 2b). The mean (SD) number of faricimab injections between baseline and month 12 was 6.9 (2.1) for previously treated eyes. These eyes received 4.5 (1.2) injections in months 1–6 and 2.4 (1.3) injections in months 7–12, respectively (Fig. 2c).

### Visual acuity outcomes

The mean (SD) VA at baseline was 63.9 (15.2) ETDRS letters for treatment-naïve and 65.8 (15.3) ETDRS letters for previously treated eyes. In total, 46.9% of treatment-naïve eyes and 53.03% of previously treated eyes had a baseline VA of ≥70 letters (Table 1).

**Fig. 3:**
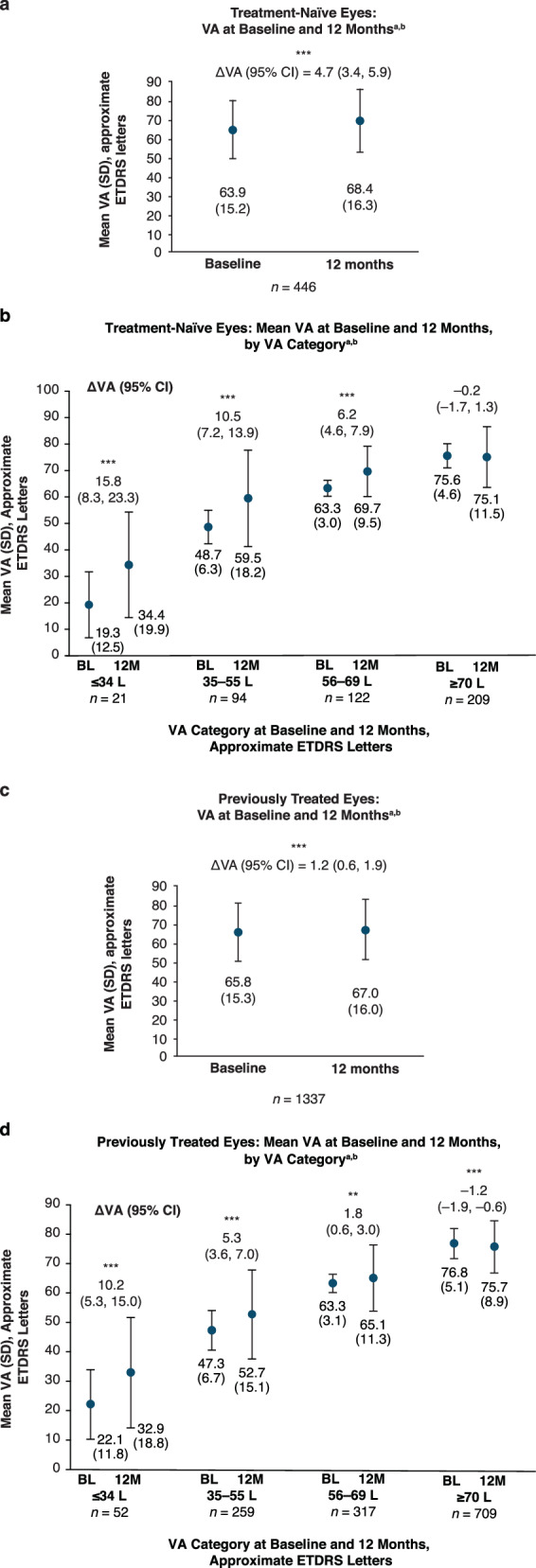
VA at 12 months since faricimab initiation for the 12-month cohort. **a** Mean VA at baseline and month 12 in treatment-naïve eyes (ETDRS letters). **b** Mean VA change from baseline to month 12 by baseline VA in treatment-naïve eyes. **c** Mean VA at baseline and month 12 in previously treated eyes. **d** Mean VA change from baseline to month 12 by baseline VA in treatment-naïve eyes. **Nominal *P* < 0.01 vs baseline. ***Nominal *P* < 0.001 vs baseline. ^a^Among eyes with VA data at baseline and 12-month timepoint. ^b^Baseline corresponds with the first faricimab injection. CI confidence interval, ETDRS Early Treatment Diabetic Retinopathy Study, L letters, SD standard deviation, VA visual acuity.

64.6% of treatment-naïve eyes had VA recorded at 12 months. For these eyes, the mean (SD) VA at 12 months was 68.4 (16.3) letters (Fig. 3a). VA change (95% CI) from baseline to 12 months was 4.7 (3.4, 5.9) letters (Fig. 3a); eyes in the lowest VA score (≤34 letters) category had the highest VA gain (15.8 [95% CI 8.3, 23.3] letters; Fig. 3b). VA change in eyes with 35–55, 56–69 and ≥70 was 10.5 (95% CI 7.2, 13.9), 6.2 (95% CI 4.6, 7.9) and −0.2 (95% CI −1.7, 1.3), respectively.

91.8% of previously treated eyes had VA recorded at 12 months. The mean (SD) VA at 12 months was 67.0 (16.0) letters (Fig. 3c). Mean VA change (95% CI) from baseline to 12 months was −1.2 (0.6, 1.9) letters (Fig. 3c). Similar to the treatment-naïve group, in previously treated eyes, eyes in the lowest VA score category (≤34 ETDRS letters) had the highest VA gains (10.2 [95% CI 5.3, 15.0] ETDRS; Fig. 3d). VA change in eyes with 35–55, 56–69 and ≥70 was 5.3 (95% CI 3.6, 7.0), 1.8 (95% CI 0.6, 3.0) and −1.2 (95% CI 1.9, 0.6), respectively. In total, 43.5% of treatment-naïve eyes with VA < 70 ETDRS letters at baseline (*n* = 237) attained driving-level VA (≥70 letters) at 12 months, and most (85.7%) of the treatment-naïve eyes with VA ≥ 70 letters at baseline (*n* = 209) were able to maintain driving vision at 12 months. In the previously treated eyes (*n* = 628 qualified for this analysis), 27.1% of eyes with VA < 70 ETDRS letters at baseline attained driving vision at 12 months, whereas the majority (85.6%) of eyes with baseline VA ≥70 letters (*n* = 709) were able to maintain it through 12 months (Supplementary Table 2).

Amongst qualifying treatment-naïve eyes, 32.1% (*n* = 143/446) gained ≥10 ETDRS letters at 12 months and 19.3% (*n* = 86/445) gained ≥15 letters; 92.0% (*n* = 404/440) avoided loss of ≥10 letters and 93.9% (*n* = 413/440) avoided loss of ≥15 letters. Amongst qualifying previously treated eyes, 16.0% (*n* = 214/1337) gained ≥10 letters at 12 months and 8.8% (*n* = 117/1332) gained ≥15 letters, and 88.7% (*n* = 1177/1327) avoided loss of ≥10 letters and 93.5% (*n* = 1241/1327) avoided loss of ≥15 letters (Supplementary Table 3).

### Safety

IOI and PIE were assessed amongst 11,139 treatment-naïve and 15,013 previously treated eyes included in the FARWIDE-nAMD/DMO studies, with any duration of follow-up since faricimab initiation. Treatment-naïve eyes received 57,641 injections and experienced 81 IOI events. The IOI rate per injection was 0.14% (95% CI 0.11%, 0.17%). Treatment-naïve eyes experienced 16 PIE events, and the PIE rate per injection was 0.03% (95% CI 0.02%, 0.05%).

Previously treated eyes received 100,741 injections and experienced 137 IOI events. The IOI rate per injection was 0.14% (95% CI 0.11%, 0.16%). These eyes experienced 44 PIE events, and the PIE rate per injection was 0.04% (95% CI 0.03%, 0.06%) (Table 2).

**Table 2:** Frequency and rate IOI and PIE (selected AEs) for eyes with nAMD and DMO.

	Treatment-naïve eyes	Previously treated eyes
Number of injections^a^	57,641	100,741
Events^a–c^		
IOI, *n*	81	137
% (95% CI)	0.14 (0.11, 0.17)	0.14 (0.11, 0.16)
PIE, *n*	16	44
% (95% CI)	0.03 (0.02, 0.05)	0.04 (0.03, 0.06)

*AE* adverse event, *CI* confidence interval, *DMO* diabetic macular oedema, *EMR* electronic medical record, *IOI* intraocular inflammation, *nAMD* neovascular age-related macular degeneration, *PIE* presumed infectious endophthalmitis, *VEGF* vascular endothelial growth factor.

^a^Amongst 11,139 treatment-naïve and 15,013 previously treated eyes meeting the eligibility criteria for safety analysis in the FARWIDE-nAMD/DMO studies. Eyes were excluded from this analysis if there was evidence of IOI or PIE in the 12 months before the first faricimab injection. AEs are included if they are within 180 days after a faricimab injection and before a subsequent anti-VEGF injection. If AEs of the same type occur within 30 days of each other, then only the first is included. Only one event per injection is counted.

^b^IOI and endophthalmitis (presumed infectious endophthalmitis) events were identified using the post-operative complications, diagnoses or clinical examination findings on the patient record in the EMR. Endophthalmitis events were additionally identified using surgical records of vitreous biopsy or anterior chamber tap or intravitreal treatments of ceftazidime or vancomycin on the patient’s record in the EMR. A detailed list of diagnoses, clinical exam findings and post-operative complications relating to IOI or endophthalmitis are provided in Supplementary Table 4.

^c^The exact χ² method was used to calculate the 95% CI for the rates.

## Discussion

FARWIDE is the largest real-world study of faricimab outside the United States, evaluating its effectiveness, safety and durability in the UK for nAMD and DMO. The FARWIDE-DMO results show that after 12 months of follow-up, VA improved by approximately 5 ETDRS letters on average in treatment-naïve eyes (mean VA [95% CI] change of 4.7 [3.4, 5.9]). Approximately 43% of eyes attained and 86% of eyes maintained driving-level VA (≥70 letters). Also, 32.1% gained two lines of vision and 19.3% gained three lines of vision at 12 months. VA remained stable in previously treated eyes. Approximately 27% of previously treated eyes attained and 86% maintained driving-level VA 12 months after faricimab initiation.

In treatment-naïve and previously treated eyes, the mean number of injections declined by almost half during the second 6 months compared with the first 6 months of follow-up, suggesting treatment interval extension following the initial loading phase. IOI and PIE rates were low and in line with those observed in phase 3 faricimab trials [15, 19] and other large real-world studies [29].

Results from FARWIDE-DMO from patients with 12 months of follow-up are consistent with the YOSEMITE/RHINE clinical trials in DMO, which demonstrated vision benefits with faricimab in treatment-naïve patients over 1 year [15]. Vision improvements in treatment-naïve eyes in the FARWIDE-DMO study were also in line with other real-world studies on patients with DMO treated with faricimab, which have shown promising results with improved outcomes over short periods of time [21–25]. A recently published study of 127 patients with nAMD and DMO from the UK showed that for eyes with DMO, after 5 months of treatment with faricimab, best-corrected VA increased from 61.1 to 72.8 letters in treatment-naïve patients [21]. The TAHOE study included 181 eyes from 136 patients and reported improvements of 2.2 letters in best-corrected VA for patients treated with faricimab in the USA [22]. Kusuhara et al. [23] reported that, in patients with DMO, faricimab treatment preserves VA while improving macular thickness in treatment-naïve patients and those previously treated with anti-VEGF in Japan. The sharp reduction in the number of injections in treatment-naïve and previously treated eyes in months 7–12 in FARWIDE-DMO suggests that faricimab has the potential to swiftly extend treatment intervals. Similar visual outcomes with treatment extension have been reported in the FARETINA-DMO study [25]. The higher number of mean injections in the first 6 months (approximately 4.5 injections in treatment-naïve and previously treated eyes) is reflective of the standard loading course of faricimab (one injection per month for 4 months) in a high proportion of eyes (61% of treatment-naïve and 48% of previously treated eyes).

Greater treatment durability is of key interest globally, especially in countries with already strained healthcare systems, such as the UK. Using a modelling approach, Li et al. [30] showed that the use of faricimab resulted in circumventing more than 12,000 treatment delays and more than £15 million (GBP/£; about $18.5 million US dollars) expenditure compared with aflibercept in a typical UK National Health Service eye hospital. Therefore, it is important to not only study the long-term safety and effectiveness outcomes of faricimab treatment, but also to assess its durability as a treatment option in the real-world setting. Sood et al. [31] reported that after 2 years faricimab is more cost-effective than prior anti-VEGF treatments. Buhrer and Diles [32] reported this time to be 3 years and suggested faricimab to be an option to address current unmet needs in the treatment of DMO. Using cost minimisation and budget impact analysis, Klabukova et al. [33] showed the use of faricimab to be a cost-effective option in Russia compared with aflibercept and ranibizumab over 2 years.

IOI and PIE rates were low in the FARWIDE study, in line with phase 3 clinical trials of faricimab [15, 19]. Similar safety results have also been shown in previous real-world studies [22, 23, 29, 34]. Similar to FARWIDE, the FARETINA study showed low rates of IOI and endophthalmitis from treatment-naïve and previously treated eyes with nAMD and DMO receiving 261,503 injections (IOI, 0.12%; endophthalmitis, 0.07%). In the TAHOE study, no cases of IOI, endophthalmitis, vasculitis or retinal artery occlusion were reported in both treatment-naïve and previously treated patients with DMO receiving 756 injections [22].

Strengths of the FARWIDE study include the large sample size and representativeness within the UK with inclusion of data from 35 National Health Service trusts. FARWIDE provides an expanded opportunity to evaluate outcomes of faricimab in previously treated eyes, the inclusion of which was limited in clinical trials. FARWIDE uses an ophthalmology EMR with structured fields to capture the ocular information more completely and in a standardised way, which leads to high-quality data.

FARWIDE has several limitations. The data capture is limited to routine clinical practice, which may lead to missing data. Although VA is described in ETDRS letters, VA measurements were not standardised. logMAR and Snellen score were also used in clinics and converted into approximate ETDRS letters for analysis. Also, the data captured in FARWIDE-DMO is limited to the UK and may not be readily generalisable to other countries or private practice in the UK. For safety events, milder AEs may not have been accurately recorded in the EMR. Finally, no anatomic data were collected, so the impact of faricimab on retinal fluid clearance in this population is not assessed.

In conclusion, results from FARWIDE-DMO add to the growing body of evidence supporting the effectiveness, safety and durability of faricimab. These results indicate clinical improvements with no new safety concerns, reinforcing the favourable risk–benefit profile of the drug. Furthermore, the data suggest that faricimab may lead to swift extension of treatment intervals in the real world, reducing the burden of treatment and making it a promising option for long-term management. These findings emphasise the potential of faricimab to effectively address unmet clinical needs in treatment of DMO. Longer-term effectiveness and safety outcomes will be evaluated in future analyses of this ongoing study.

## Summary

### What was known before

- Faricimab is a novel bispecific antibody targeting two separate pathways inhibiting both vascular endothelial growth factor-A and angiopoietin-2. Faricimab has demonstrated vision gains and improved anatomical outcomes in patients with diabetic macular oedema in phase 3 clinical trials. By targeting both pathways, faricimab has the potential to reduce treatment burden by extending the injection interval compared with standard anti–vascular endothelial growth factor therapies.
- Clinical trials are conducted under strict protocols with carefully selected patient populations, standardised treatment regimens and rigorous follow-up schedules. In contrast, real-world settings include more diverse patient populations with varying disease severity, treatment adherence and healthcare access. These factors could lead to differences in treatment effectiveness, safety and durability of response when faricimab is used in routine clinical practice.
- Studying real-world outcomes is essential for understanding the true impact of faricimab in everyday clinical practice. Real-world evidence provides insight into safety, effectiveness, treatment adherence and injection frequency outside the controlled clinical trial environment. Real-world studies also complement clinical trial findings by capturing data on broader patient populations and practical aspects of treatment administration.

### What this study adds

- FARWIDE-DMO is a real-world retrospective study using data from 35 National Health Service sites in the United Kingdom and focusses on evaluating the effectiveness, safety and durability of faricimab.
- The results of the FARWIDE-DMO study from patients with 12 months of follow-up after their first faricimab injection shows that treatment-naïve eyes had improved vision from baseline, whereas eyes that had previously received anti–vascular endothelial growth factor treatment(s) had stable vision from baseline at 12 months. Injection frequency was lower in the second 6 months of treatment versus the first 6 months for both treatment-naïve and previously treated eyes, and rates of intraocular inflammation and potentially infectious endophthalmitis were similar to those in phase 3 clinical trials of faricimab.
- FARWIDE-DMO provides real-world evidence on the effectiveness and safety of faricimab while also demonstrating a reduction in injection number after the first 6 months of treatment, suggesting that faricimab may be a durable treatment for DMO, potentially decreasing the treatment burden of diabetic macular oedema.

## Supplementary information

Supplementary information

## Data Availability

The participating sites did not give written consent for disaggregated data to be shared, so raw data must remain confidential and cannot be made available. Aggregated data are available upon reasonable request.
