# Supplementary material for: Real-world treatment patterns and visual outcomes of faricimab in patients with diabetic macular oedema in the UK at 12 months: the FARWIDE-DMO study
Source: Eye (Lond). 2025 Oct 23;39(18):3350–8. doi: 10.1038/s41433-025-04059-8 (PMC12669761; doi:10.1038/s41433-025-04059-8)
Supplement: Supplementary file 1 — Supplementary information [file 41433_2025_4059_MOESM1_ESM.docx]

# Supplement

This supplement includes additional details on methods as well as supplementary tables providing expanded data on select results and methods. This section supports the main text by providing further details into the study’s analyses and findings.

# Supplementary Methods

## Data source

Medisoft (Medisoft Ltd., Leeds, UK) electronic medical records are used to record ophthalmic assessment, treatment and diagnostic information at more than 200 hospitals in the United Kingdom and are designed to capture highly structured clinical data which allows for the collation of data from multiple sites to determine real-world clinical outcomes.

## Index of Multiple Deprivation

Socioeconomic status was defined by the Index of Multiple Deprivation (IMD). The IMD is a small area measure of relative deprivation across the constituent nations of the United Kingdom. Areas are ranked from most deprived (1) to least deprived (10). The unit areas used to calculate the IMD are Lower layer Super Output Areas for England (Consumer Data Research Centre, 2020). In Scotland, the unit areas used were Data Zones (Scottish Government, 2020). The deciles are calculated by ranking the 32 844 LSOAs in England and 6976 Data Zones in Scotland. Areas in England and Scotland are ranked separately. The deciles are calculated by ranking the 32 844 LSOAs in England and 6976 Data Zones in Scotland. Areas in England and Scotland are ranked separately. IMDs were categorised into decile categories 1–2, 3–4, 5–6, 7–8 and 9–10.

## Additional visual acuity outcomes

The semi-quantitative values of count fingers, hand motion, perception of light and no perception of light was converted to 0 Early Treatment Diabetic Retinopathy Study letter score for the purposes of the analysis (Norridge et al., 2023) as are any logMAR values worse than 1.68. A correction priority order was implemented to reduce potential bias if a different correction was used at baseline and follow-up intervals for any given patient. At each time point, the best value between habitual correction visual acuity (e.g. glasses, contact lens, refraction test) and unaided visual acuity was used, and where no results are available for either of these measures, pinhole visual acuity was used (Jaycock et al., 2009).

## Adverse event analysis

Intraocular inflammation and presumed infectious endophthalmitis events were identified using the post-operative complications, diagnoses or clinical examination findings on a patient’s electronic medical records. Any endophthalmitis was excluded from intraocular inflammation. The full list of clinical terms and procedures used to identify intraocular inflammation and presumed infectious endophthalmitis are detailed in Supplementary Table 4.

Rates were evaluated for eyes with neovascular age-related macular degeneration and diabetic macular oedema eyes in FARWIDE with any duration of follow-up since baseline with no evidence of IOI or PIE in the 12 months before the first faricimab injection. This was done to ensure that all available safety data on faricimab from the FARWIDE study was reported, rather than safety data from select subpopulations only. Study eyes were observed for adverse events from the date of the first faricimab injection (baseline) until the study eye received an anti–vascular endothelial growth factor injection other than faricimab or until the date of data extraction if the former did not occur. All events occurring during this time were evaluated.

## Statistical analysis

Data were summarised descriptively and expressed as mean (standard deviation) for continuous variables or as the number of eyes/patients (n) (%) for categorical variables. Mean changes in visual acuity (Early Treatment Diabetic Retinopathy Study letters) corresponding 95% confidence intervals were analysed using generalised estimating equations. Nominal *P* values were calculated as appropriate. As *P* values are nominal and not adjusted for multiplicity, no formal statistical conclusion should be made based on these. Results were analysed separately for treatment-naïve and previously treated eyes. R statistical software was used to perform the analyses. Mean (standard deviation) was reported for continuous variables. 95% confidence intervals for the adverse event rate were evaluated using the exact χ² method.

**References**

Consumer Data Research Centre. Index of Multiple Deprivation (IMD) 2020 [Available from: https://data.cdrc.ac.uk/dataset/index-multiple-deprivation-imd.

Scottish Government. Scottish Index of Multiple Deprivation (SIMD) 2020 [Available from: https://www.spatialdata.gov.scot/geonetwork/srv/eng/catalog.search#/metadata/02866b0b-66e5-46ab-9b1c-d433dc3c2fae.

Norridge CFE, Gruszka-Goh MH, McKibbin M, Henry P, Donachie J. National Ophthalmology Database Audit: the first report of age-related macular degeneration audit (AMD) 2023 [Available from: <https://nodaudit.org.uk/sites/default/files/2023-02/NOD%20AMD%20Audit%20Full%20Annual%20Report%202023_0.pdf>].

Jaycock P, Johnston RL, Taylor H, Adams M, Tole DM, Galloway P, et al. The Cataract National Dataset electronic multi-centre audit of 55,567 operations: updating benchmark standards of care in the United Kingdom and internationally. Eye (Lond). 2009;23(1):38–49.

## **Supplementary Table 1.** List of the FARWIDE-DMO participating study sites.

| **Participating NHS trusts** |
| --- |
| Barking, Havering and Redbridge University Hospitals NHS Trust |
| Bedford Hospital NHS Trust |
| Bradford Teaching Hospitals NHS Foundation Trust |
| Buckinghamshire Healthcare NHS Trust |
| Calderdale and Huddersfield NHS Foundation Trust |
| Colchester Hospital University NHS Foundation Trust |
| East Cheshire NHS Trust |
| East Sussex Healthcare NHS Trust |
| Gloucestershire Hospitals NHS Foundation Trust |
| Great Western Hospitals NHS Foundation Trust |
| Hull and East Yorkshire Hospitals NHS Trust |
| Isle of Wight NHS Trust |
| Leeds Teaching Hospitals NHS Trust |
| Liverpool University Hospitals NHS Foundation Trust |
| London North West Healthcare NHS Trust |
| Mid Cheshire Hospitals NHS Foundation Trust |
| Mid Yorkshire Hospitals NHS Trust |
| NHS Grampian |
| NHS Highland |
| Oxford University Hospitals NHS Foundation Trust |
| Royal Berkshire NHS Foundation Trust |
| Royal Cornwall Hospitals NHS Trust |
| Royal Free London NHS Foundation Trust |
| Salisbury NHS Foundation Trust |
| Sheffield Teaching Hospital NHS Foundation Trust |
| South Tees Hospitals NHS Foundation Trust |
| South Warwickshire NHS Foundation Trust |
| Taunton and Somerset NHS Foundation Trust |
| The Hillingdon Hospital NHS Foundation Trust |
| The Newcastle Upon Tyne Hospitals NHS Foundation Trust |
| Torbay and South Devon NHS Foundation Trust |
| University Hospital Southampton NHS Trust |
| University Hospitals Bristol NHS Foundation Trust |
| Warrington and Halton Hospitals NHS Foundation Trust |
| Wrightington, Wigan and Leigh NHS Foundation Trust |

*NHS* National Health Service.

## **Supplementary Table 2.** Patient-eyes gaining and maintaining VA ≥70 ETDRS letters at 12 months in the FARWIDE-DMO 12-month cohort.

| **Treatment-naïve patient-eyes** | **Qualifying eyes^a^, *n*** | **Attaining or maintaining eyes, *n* (%)** |
| --- | --- | --- |
| **Attainment of ≥70 ETDRS letters** | | |
| **VA <70 ETDRS letters at baseline** | 237 | 103 (43.5) |
| VA ≤34 ETDRS letters at baseline | 21 | 0 (0) |
| VA 35–55 ETDRS letters at baseline | 94 | 31 (33.0) |
| VA 56–69 ETDRS letters at baseline | 122 | 72 (59.0) |
| **Maintenance of VA ≥70 ETDRS letters, n (%)** | | |
| VA ≥70 ETDRS letters at baseline | 209 | 179 (85.7) |
| **Previously treated patient-eyes** | **Qualifying eyes, n** | **Attaining or maintaining eyes, n (%)** |
| **Attainment of ≥70 ETDRS letters** | | |
| **VA <70 ETDRS letters at baseline** | 628 | 170 (27.1) |
| ≤34 ETDRS letters at baseline | 52 | 2 (3.9) |
| 35–55 ETDRS letters at baseline | 259 | 40 (15.4) |
| 56–69 ETDRS letters at baseline | 317 | 128 (40.4) |
| **Maintenance of ≥70 ETDRS letters, *n* (%)** | | |
| VA ≥70 ETDRS letters at baseline | 709 | 607 (85.6) |

^a^Eyes qualifying for attainment of ≥70 ETDRS letters had a VA of <70 letters at baseline. Eyes qualifying for maintenance of ≥70 ETDRS letters had a VA of ≥70 ETDRS letters at baseline.

*ETDRS* Early Treatment Diabetic Retinopathy Study, *nAMD* neovascular age-related macular degeneration, *VA* visual acuity.

## **Supplementary Table 3.** Patient-eyes gaining and losing ≥10 ETDRS letters at 12 months in the FARWIDE-DMO 12-month cohort.

| **Treatment-naïve patient-eyes** | **Qualifying eyes, *n*** | **Eyes gaining/avoiding loss of VA, *n* (%)** |
| --- | --- | --- |
| Gain of ≥10 ETDRS letters VA | 446^a^ | 143 (32.1) |
| Gain of ≥15 ETDRS letters VA | 445^b^ | 86 (19.3) |
| Avoiding loss of ≥10 ETDRS letters VA | 440^c^ | 404 (91.8) |
| Avoiding loss of ≥15 ETDRS letters VA | 440^d^ | 413 (93.9) |
| **Previously treated patient-eyes** | **Qualifying eyes, *n*** | **Eyes gaining/losing VA, *n* (%)** |
| Gain of ≥10 ETDRS letters VA | 1337^a^ | 214 (16.0) |
| Gain of ≥15 ETDRS letters VA | 1332^c^ | 117 (8.8) |
| Avoiding loss of ≥10 ETDRS letters VA | 1327^c^ | 1177 (88.7) |
| Avoiding loss of ≥15 ETDRS letters VA | 1327^d^ | 1241 (93.5) |

^a^ Eyes with ≤90 ETDRS letters at baseline. ^b^ Eyes with ≤85 ETDRS letters at baseline. ^c^ Eyes with VA of ≥10 letters at baseline. ^d^ Eyes with ≥15 letters at baseline. Percentage of eyes avoiding loss of ≥10 or ≥15 ETDRS letters is defined as ([total qualifying eyes – eyes losing vision]/qualifying eyes) × 100. *DMO* diabetic macular oedema, *ETDRS* Early Treatment Diabetic Retinopathy Study, *VA* visual acuity.

## **Supplementary Table 4.** Diagnoses, clinical examination findings, post-operative complications and procedures used to identify IOI and PIE following an injection.

| **Diagnoses and clinical examination findings (IOI)** | **Associated ICD-10 code** |
| --- | --- |
| AC cells 0.5+; AC cells 1+; AC cells 2+; AC cells 3+; AC cells 4+; acute anterior uveitis; anterior uveitis; iritis | H20.0 |
| Chronic anterior uveitis | H20.1 |
| Drug-induced uveitis; fibrinous uveitis; idiopathic uveitis; intermediate uveitis; iridocyclitis; post-operative uveitis; uveitis; vitritis | H20.9 |
| Choroiditis involving the macula; retinitis involving the macula | H30.0 |
| Diffuse choroiditis | H30.1 |
| Pars planitis | H30.2 |
| Chorioretinitis; chorioretinitis involving the macula; choroiditis; posterior uveitis; retinitis | H30.9 |
| Perivascular infiltrate; retinal vasculitis; retinal vasculitis – mixed arteritis and periphlebitis; retinal vasculitis – non-occlusive; retinal vasculitis – occlusive; retinal vasculitis –periphlebitis / venous sheathing; retinal vasculitis – predominantly arteritis; vascular sheathing | H35.0 |
| 1+ vitreous inflammation; 2+ vitreous inflammation; 3+ vitreous inflammation; 4+ vitreous inflammation; trace vitreous inflammation; vitreous cells present; vitreous haze 0.5+; vitreous haze 1+; vitreous haze 2+; vitreous haze 3+; vitreous haze 4+; vitreous inflammation; vitreous inflammation –no red reflex; vitreous inflammation – red reflex present | H43.8 |
| Panuveitis | H44.1 |
| **Post-operative complications (IOI)** | **Associated ICD-10 code** |
| anterior uveitis; post-operative uveitis; retinal vasculitis; vitritis | N/A |
| **Diagnoses (PIE)** | **Associated ICD-10 code** |
| 1 mm hypopyon; 2 mm hypopyon; 3 mm hypopyon; 4 mm hypopyon; AC cells 0.5+; AC cells 1+; AC cells 2+; AC cells 3+; AC cells 4+; hypopyon; trace hypopyon | H20.0 |
| Secondary open angle glaucoma (acute anterior uveitis) | H40.4 + H20.0 |
| Secondary open angle glaucoma (panuveitis) | H40.4 + H44.1 |
| Post-operative endophthalmitis | H44.0 |
| Endophthalmitis; exogenous fungal endophthalmitis; panuveitis | H44.1 |
| **Post-operative complications (PIE)** | **Associated ICD-10 code** |
| Endophthalmitis; hypopyon | N/A |
| **Procedures (PIE)** | **Associated OPCS-4 code** |
| Anterior chamber tap | C69.8 |
| Intravitreal injection (specified agents: amikacin 0.4 mg + vancomycin 1.0 mg in 0.2 ml; ceftazidime 2.2 mg/0.1 ml; ceftazidime 2.25 mg/0.1 ml; vancomycin 1 mg/0.1 ml; vancomycin 2 mg/0.1 ml; vancomycin 5 mg/0.5 ml) | C79.4 |
| Vitreous biopsy | C79.8 |

There are some terms in the Medisoft library (AC cells, panuveitis) which, if recorded on their own, could infer that either an IOI or a PIE event occurred. These terms were assumed to indicate that an IOI event occurred unless recorded alongside another PIE term.

*AC* anterior chamber, *IOI* intraocular inflammation, *N/A*, not applicable; *PIE* presumed infectious endophthalmitis.
